# Inner Dialogues and Nutritional Anxiety in Sports Tourism: Understanding Runners’ Habits in Pre-Race Food-Related Stress Abroad

**DOI:** 10.3390/nu17172817

**Published:** 2025-08-29

**Authors:** Mateusz Rozmiarek

**Affiliations:** Department of Sports Tourism, Faculty of Physical Culture Sciences, Poznan University of Physical Education, 61-871 Poznan, Poland; rozmiarek@awf.poznan.pl

**Keywords:** sports tourism, sport, tourism, running, inner dialogue, anxiety, nutrition, half marathon, Poznan, Poland

## Abstract

**Background/Objectives**: For runners competing abroad in sports events, the hours before a race are marked by heightened psychological tension, where even food choices can feel crucial to success. While pre-race nutrition is often addressed in terms of physiological needs, little is known about the inner psychological processes that accompany food decisions in unfamiliar cultural and environmental contexts. This study explores the inner dialogues, anxieties, and coping mechanisms of international runners facing the question of whether and what to eat before competition. **Methods**: A qualitative study was conducted with twelve international participants (from the United Kingdom, Germany, and Ukraine) of the Poznan Half Marathon 2025. Data were collected through semi-structured in-depth interviews. Participants possessed a minimum of two years’ experience competing in international events. **Results**: Three thematic areas were identified: (1) anticipatory anxiety and fear of making nutritional mistakes before the race, (2) internal negotiation between prior nutritional knowledge and situational trust, and (3) ritualization and individualized norms as fundamental mechanisms of psychological regulation. These themes influenced how runners experienced pre-race nutrition, shaping their emotional states, decision-making processes, and coping strategies in the context of international competition. **Conclusions**: Pre-race nutrition decisions are deeply embedded in emotional and cognitive landscapes shaped by stress, cultural context, and individual history. Recognizing these inner dynamics can help coaches, sports nutritionists, and event organizers better support the psychological well-being of traveling athletes.

## 1. Introduction

In the past three decades, there has been a dynamic increase in interest in sports tourism, where physically active individuals integrate competitive sports with leisure travel [1,2,3]. A particularly visible trend is the development of running tourism, where international marathons, half-marathons, and city races attract thousands of participants from different countries [4,5,6,7]. For many amateur runners, participation in a race abroad becomes not only a sporting goal but also a form of self-actualization, cultural exploration, and shared community experience [8,9]. Such events are characterized by high emotional intensity and considerable organizational demands—participants must prepare not only physically but also face the logistics of travel, changes in climate, time zones, and cultural environments, and adapt to unfamiliar surroundings. Runners, often highly committed to training and competition, although motivated by various reasons for racing [10,11,12,13,14], are confronted with the necessity of making numerous decisions in unfamiliar conditions, which makes their experience particularly interesting from an interdisciplinary research perspective—encompassing sport, tourism, psychology, and nutrition.

While stress in sports tourism has been examined only sporadically—with studies addressing, for example, thermal stress in major events [15] and the potential of sport tourism activities to reduce general stress [16], with some evidence suggesting that aspects of the experiencescape, such as social and environmental factors, influence psychological recovery [17]—nutrition-specific psychological strain has not been studied extensively. However, there are emerging works focusing on nutritional challenges faced by active sports tourists [18] and on runners’ experience of local food customs during their travels [19]. Additionally, some research has explored how nutrition contributes to preserving the health and physical condition of sports volunteers working at international events [20,21]. Given these diverse but still limited insights, it is therefore necessary to consider nutrition-related psychological strain alongside other known stressors to identify where intervention and support might be most effective.

Proper pre-race nutrition is a key component of runners’ preparation, affecting endurance, recovery, and overall performance readiness [22,23,24]. Dietary recommendations for endurance athletes emphasize the importance of adequate carbohydrate intake, hydration, and avoiding foods that may cause gastrointestinal distress [25]. However, putting these guidelines into practice, especially in the context of international travel, comes with numerous challenges [18]. Choosing appropriate food in a foreign country becomes, for many runners, a risk-laden decision that goes beyond caloric calculations. For some, the issue is not merely the availability of familiar products, but also uncertainty regarding the ingredients of dishes, hygiene standards, or even local customs related to food preparation and serving. As a result, a specific form of psychological strain may arise—nutritional anxiety—which is distinct from general pre-competition anxiety and refers to worries and tensions directly linked to food-related decisions before a race. In many cases, this nutritional anxiety coexists with, but is not reducible to, broader anticipatory stress about the event itself.

Studies in gastroenterology indicate strong links between stress and gastrointestinal functioning in athletes [26,27], particularly in endurance sports such as long-distance running [28]. Psychological stress can lead to disturbances in gastrointestinal motility, increased intestinal permeability, and dysbiosis of the gut microbiota, all of which contribute to gastrointestinal complaints—one of the most common issues faced by runners during competition [29,30]. Nutritional anxiety may further exacerbate these issues by heightening vigilance and physiological arousal specifically in relation to eating, thus adding a distinct pathway by which stress impacts gastrointestinal function. This phenomenon complicates nutrition management during travel, where the stress of competition is compounded by the pressure of adapting to unfamiliar environments. At the same time, subjective experiences and coping strategies are crucial for maintaining optimal performance and well-being. The literature emphasizes that psychological mechanisms of control and perceived safety—such as meal planning, risk avoidance, and the search for familiar foods—can help mitigate the negative impact of stress on gastrointestinal function and athletic performance [31]. However, understanding these processes within the broader context of race preparation and everyday nutritional practices—especially in situations that require psychophysical adaptation—remains limited, highlighting the need for research that integrates biological, psychological, and cultural perspectives.

Despite growing interest in sports nutrition and the increasing number of studies analyzing the impact of stress on athlete functioning, there remains a lack of comprehensive work that bridges these two domains in the context of sports tourism, particularly among runners competing internationally. Moreover, much of the existing literature approaches sports nutrition primarily as a technical domain, focusing on measurable parameters such as macronutrient intake [32,33,34], supplementation protocols [35], or hydration strategies [36,37], without adequately addressing the lived experiences, psychological dimensions, and anxieties directly tied to eating in competitive contexts. Meanwhile, international travel introduces additional challenges—from adapting to unfamiliar culinary cultures and limited access to known products, to the pressure of avoiding mistakes that could negatively affect performance. This complex dynamic calls for the inclusion of athletes’ subjective experiences and inner dialogues related to food, which remain underexplored in the current literature. Studying these internal dialogues is critical because they reveal the cognitive and emotional processes that precede and influence observable behaviors and physiological outcomes, providing a more nuanced understanding of how athletes negotiate nutritional challenges in real-time. This focus allows to capture the dynamic interplay between thought patterns and coping strategies that may not be evident through behavioral observation alone. From a theoretical perspective, pre-race nutritional stress can be framed through established models of stress and health behavior, highlighting how cognitive appraisal, perceived risk, and motivational factors interact to shape athletes’ decisions in novel environments. Pre-race nutritional stress is particularly salient because it directly links cognitive appraisal, physiological vulnerability, and behavioral decisions in a high-stakes context, creating measurable consequences for both performance and well-being. Studying these food-related inner dialogues therefore provides unique insights into stress regulation mechanisms that may not be as directly observable through other situational challenges encountered by athletes during international competitions [38]. In particular, there is a need for qualitative studies that could illuminate how runners cope with tension and uncertainty in this unique context, and what strategies they adopt to minimize nutrition-related stress in unfamiliar environments. Such insights could inform targeted interventions by sport psychologists, nutritionists, and event organizers, contributing to more effective preparation protocols and athlete support systems. Filling this gap is crucial not only for advancing scientific knowledge but also for developing practical recommendations that support athletes in effectively managing their nutrition and emotions during international competitions.

Therefore, the aim of the present study is to explore the internal dialogues, anxieties, and coping strategies related to pre-race nutrition among amateur runners participating in international sporting events. The analysis focuses on the psychological and behavioral mechanisms that shape food-related choices in the pre-competition period and on how these mechanisms influence athletes’ subjective experiences.

## 2. Materials and Methods

This research employed a qualitative approach, centering on the use of in-depth interviews (IDIs) as the primary data collection method [39]. IDIs are widely recognized in the methodological literature as a valuable means of accessing detailed, personal insights and for examining complex, multifaceted issues [40]. Unlike group-based techniques such as focus groups, the one-on-one interview format allows for more refined and individualized data, often encouraging participants to speak more freely and share deeper reflections—especially when the subject matter involves personal or sensitive experiences [41]. Moreover, the qualitative method was selected to capture these complex, subjective experiences that cannot be fully understood through quantitative or physiological measures. It enables a more detailed insight into how cultural adaptation and emotional coping shape runners’ nutrition-related stress before races abroad.

### 2.1. Sample Selection

The study included twelve international runners—eight men and four women—who participated in the Poznan Half Marathon 2025. The participants were equally divided among three national groups: four from the United Kingdom, four from Germany, and four from Ukraine. Each individual had at least two years of experience traveling abroad for running competitions, which qualified them as active sports tourists. Table 1 presents anonymized participant aliases along with their gender and country of residence.

The research focused specifically on pre-race nutrition-related decision-making and psychological coping strategies, as part of a wider exploration of the broader sports tourism experience, encompassing elements such as travel logistics, dietary practices, and physical activity undertaken in international contexts. The study did not specifically assess whether participants had received formal training in nutrition or had obtained professional dietary advice prior to the race. Nutritional decisions discussed in the interviews reflected the participants’ personal experiences and practices. All participants were legally adults. The sample was selected using purposive sampling, guided by two inclusion criteria: (1) permanent residence outside of Poland and travel to Poznan with the explicit purpose of participating in the half marathon; and (2) a minimum of two years’ experience in international sports-related travel. To improve recruitment of eligible participants and capture a wider variety of information-rich cases, the purposive sampling method was supplemented with a snowball sampling approach following Patton’s recommendations [42], where initial respondents referred other suitable runners for the study. Individuals under the legal age or not meeting these requirements were excluded from participation.

### 2.2. Justification for Sample Size

The number of participants was determined in line with qualitative research conventions, which emphasize reaching data saturation as the primary criterion for finalizing sample size. Saturation occurs when additional interviews no longer produce novel information or meaningful patterns relevant to the research objectives. In the context of this study, saturation was reached after twelve interviews, indicating that the collected data were sufficient to provide a thorough and nuanced understanding of the subject matter. This sample size is consistent with widely accepted guidelines in qualitative methodology [43,44]. While relatively small, the sample was appropriate for the exploratory aims of the study, facilitating the identification of context-specific themes and subtle psychological dynamics that would be difficult to uncover through quantitative approaches.

Importantly, saturation was evaluated not solely based on the repetition of themes, but also by considering the diversity and variability across individual cases. The dataset was examined for multidimensionality to ensure that the identified themes captured a broad spectrum of perspectives rather than mere thematic redundancy. This process involved assessing the richness and subtle differences in coding among participants, as well as determining whether subsequent interviews continued to enhance the explanatory depth of the coding framework.

### 2.3. Procedure

The study employed semi-structured interviews, a method that facilitated open exploration of participants’ perspectives through flexible, open-ended questioning. All interviews were conducted in English throughout April 2025, with each session averaging approximately one hour in duration. Audio recordings were made only after securing explicit consent from each participant. The research adhered strictly to recognized ethical standards for scientific inquiry. Participants were fully briefed on the purpose and scope of the study, the procedures involved, and their right to withdraw at any time without consequence. Informed consent was obtained for both participation and the recording of interviews. Anonymity and confidentiality were rigorously maintained, and all findings were presented in a manner that ensured the non-identifiability of individuals.

To enhance methodological consistency, a single researcher conducted all interviews. This approach ensured uniformity in interview style and contributed to building trust and rapport, which in turn encouraged participants to share more openly. The use of a semi-structured protocol, grounded in a pre-defined set of core questions, supported thematic comparability while allowing the flexibility to pursue personal narratives and context-specific insights in greater depth.

### 2.4. Data Analysis

The interview data underwent analysis through a systematic, four-stage thematic procedure. This process began with the transcription of audio recordings and an in-depth familiarization with the content. In the second stage, participant statements were categorized, and recurring motifs were identified. These were then grouped into broader thematic clusters, forming the basis for stage three. The final phase involved interpreting the data and deriving conclusions from the overarching themes that emerged. All steps adhered to recognized qualitative research protocols, ensuring both the reliability and interpretive validity of the findings.

The analysis was guided by Braun and Clarke’s [45] influential model of thematic analysis, which provides a structured and transparent framework for identifying and organizing patterns within qualitative data. Applying this model helped ensure the analytic process remained rigorous and reproducible.

To further strengthen the depth and credibility of the interpretation, an iterative, multi-phase strategy was adopted. Following transcription, the interview texts were reviewed several times to develop a rich understanding of participant narratives. Coding occurred in two distinct stages: an initial open coding phase that aimed to capture meaningful units of data, followed by axial coding, which served to link related codes and uncover central thematic patterns. The entire process was supported by NVivo software (version 12), which enabled efficient organization and cross-referencing of codes.

All analysis was performed by the same researcher who conducted the interviews, ensuring continuity and a deep contextual awareness of the data. This consistency facilitated the nuanced interpretation of responses and contributed to the coherence of the thematic structure. Due to the exploratory and interpretive approach of this study, formal inter-coder reliability assessments or peer reviews were not conducted. Instead, methodological rigor was ensured through a reflexive thematic analysis process, complemented by a comprehensive reflective journal that tracked coding choices, emerging insights, and theoretical considerations. To reduce confirmation bias and strengthen reflexivity, instances of analytical ambiguity, differing interpretations, and theoretical influences were systematically documented, establishing an internal audit trail that provided transparency and accountability throughout the inductive analysis. Final codes were critically reviewed, refined, and compared to ensure a robust and internally consistent analytical framework. The emerging results were contextualized through engagement with the limited but relevant academic literature, allowing for a richer interpretation of the findings. A reflexive, methodical approach was maintained throughout, with continuous cross-referencing of themes against original data segments to enhance interpretive objectivity and analytic transparency.

## 3. Results

The analysis of in-depth interviews revealed a complex and multidimensional picture of how international runners approached nutrition in the lead-up to the Poznan Half Marathon 2025. Participants’ narratives highlighted not only practical aspects of dietary choices but also deeply embedded emotional, psychological, and situational dynamics. Thematic analysis identified three overarching patterns that structure this section: (1) anticipatory anxiety and fear of making nutritional mistakes before the race, (2) the internal negotiation between prior nutritional knowledge and situational trust, and (3) the ritualization of eating behaviors and adherence to personalized food norms as mechanisms of psychological regulation. These interrelated themes illustrate that pre-race nutrition is not merely a matter of physiological preparation, but also serves as a coping strategy for dealing with uncertainty, control, and performance pressure in a foreign environment. The following sections explore each theme in detail, supported by direct quotations from the runners themselves.

### 3.1. Anticipatory Anxiety and Fear of Making Nutritional Mistakes Before the Race

Participants emphasized that the pre-race period was associated with intense stress and concern about choosing the right meals, which impacted their performance and well-being during the run. This tension was linked not only to physiological needs but also to anticipatory anxiety about making a nutritional mistake that could negatively affect their results or health. The conversations revealed an image of internal struggle and unease related to race readiness, where food functioned as a means of exerting control within the complex context of pre-race preparations.

Some participants noted that the day before the race involved constant rumination over the choice of appropriate food. R1 described this state as an obsessive preoccupation with every meal: “The day before the race, I obsessively think about what to eat. Because if I make a mistake, it could cost me many valuable seconds or minutes”. His words illustrate that pre-race nutrition is not only a physiological issue but also a significant psychological burden, where each meal symbolizes risk, and the fear of “ruining” the outcome heavily influences the entire preparation. Similar emotions were expressed by R8, who planned her meals with great care to avoid surprises: “I plan my meals before the race because I don’t want any surprises,” and “Eating unfamiliar food is always risky, especially abroad”. This quote highlights a strong need for control and limiting uncertainty, and the fear of consuming unknown food amplifies the stress related to preparations. R10 also emphasized the importance of routine by avoiding experiments before the competition: “I came here to run. When it comes to food, I try to stick to what I know”.

Participants also indicated that the fear of making the wrong choice could paralyze their ability to make decisions, even when they felt hungry. R4 admitted, “Sometimes the fear of making a bad choice is overwhelming”. Anticipatory anxiety can therefore lead to hesitation and additional stress, making the simple act of eating difficult. R5 noted that the pressure to control was not only about the physical aspect of digestion but also had a psychological dimension: “Pre-race food stress isn’t just about digestion. It’s about feeling in control in an unfamiliar place”. In this way, food serves as an emotional stabilizer, and maintaining familiar habits helps sustain a sense of safety in an unfamiliar environment. Finally, R12 described this anxiety as a persistent inner voice: “There are moments when my inner voice tells me to be cautious”. This subtle dimension of fear accompanies athletes and often remains invisible, though it influences their behavior.

The findings indicate that anticipatory anxiety and the fear of making a “wrong” nutritional choice before the race are multidimensional and deeply rooted in the runners’ psyche. They relate both to concerns about physiological consequences and the desire to maintain control and safety in an unfamiliar environment.

### 3.2. Internal Negotiation Between Prior Nutritional Knowledge and Situational Trust

Participants described the decision-making process regarding nutrition before the race as neither simple nor straightforward—it required ongoing internal negotiation between their existing nutritional knowledge and trust in the conditions they encountered on site. Runners had to balance tried-and-tested routines developed during training with the necessity to adapt to local realities, which often created a conflict between a rational plan and an intuitive adjustment to the situation.

R6 expressed this state as follows: “I know what my body needs, but sometimes I have to trust the local options because I can’t carry everything with me”. His words indicate that despite a strong nutritional awareness and clear preferences, being in a foreign country demands flexibility and the ability to compromise. Similarly, R4 described how her planning was accompanied by constant monitoring of available options: “I prepare my meals in advance, even before arriving in the country where the race takes place, but I am ready to make changes if something unexpected comes up,” and “I try to make the best choice using the options available”. This shows that nutritional decisions are not merely the result of a rigid plan, but rather a dialogue between knowledge and adaptation. Meanwhile, R9 emphasized the importance of trusting his own body, which helped him manage uncertainty: “I listen to my body more than any plan. Sometimes it tells me to be careful, sometimes to try something”. This quote illustrates the dynamic nature of decision-making, where intuition and attentiveness to bodily signals play a key role. Thus, the internal negotiations encompassed both solid knowledge and sensitivity to changing circumstances.

Some participants highlighted moments when trust in local options and their own choices enhanced their sense of comfort and security. R1 noted, “When I find a familiar dish, I immediately feel more confident”. This underscores the importance of stabilizing elements in the environment that facilitate the negotiation between the plan and reality. R10 added, “Sometimes it’s about trusting yourself and believing that the choice was good enough”. This quote reflects maturity and flexibility in the approach to nutrition, which helps reduce stress and uncertainty. Finally, R8 summarized this dynamic situation with the words: “It’s like a conversation inside me—between what I know and what I feel is possible here,” and “Finding the balance is the challenge”. This metaphor aptly captures the nature of the decision-making process as an ongoing dialogue, influencing how runners cope with nutritional challenges while traveling.

The findings indicate that the negotiation between knowledge and situational trust is an integral part of pre-race nutritional choices. It is a dynamic and individual process that combines rational planning with flexibility and attentiveness to local conditions and signals from one’s own body.

### 3.3. Ritualization and Individualized Norms as Fundamental Mechanisms of Psychological Regulation

For many, in the face of uncertainty and pre-race stress, their own private rituals and individual rules regarding nutrition played a key role. Creating personal guidelines and repetitive practices allowed runners to gain a sense of control and stability, which directly impacted their mental comfort and confidence before the race. These actions did not necessarily have to be complicated—often, simply sticking to familiar dishes or maintaining certain routines was enough to ease anxiety.

R2 described the importance of such behaviors: “I always eat the same breakfast before a race—it’s my little ritual. No matter where I am, it grounds me and calms my nerves”. His words show how powerful repetition can be in building inner calm and control. This ritual becomes more than just eating—it is a part of mental preparation that stabilizes emotions. R6 added: “I have a set of personal food rules—no new foods, and always hydration in a certain way. It’s my way to avoid surprises and stay focused”. This quote emphasizes that individual rules help reduce risk and chaos, creating a safe pre-race space.

R11 highlighted how rituals influence the sense of self-efficacy, stating, “Sticking to my regular eating habits makes me feel prepared and ready” and adding, “Then I know I’ve done everything I could”. This statement shows that habits serve as a psychological anchor, reinforcing the belief in one’s readiness and determination. Some participants also emphasized that such rituals help reduce the chaos stemming from unfamiliar local conditions. R3 mentioned: “When I stick to my nutrition rules, even in a foreign country, I feel almost no risk”. Meanwhile, R7 noted that eating rituals also have an emotional dimension: “It gives me a significant sense of comfort”. This suggests that following personal dietary habits and repeating familiar eating routines can act as regulatory mechanisms that reduce psychological arousal and increase cognitive readiness in the context of competition.

The findings clearly show that ritualizing eating behaviors and adhering to personal food rules play an important role in maintaining a psychological sense of control before the start. They help runners cope with stress and uncertainty by creating a safe framework in which they feel prepared and confident. Thanks to these rituals, food becomes not only a source of energy but also a foundation of emotional stability.

## 4. Discussion

This study sheds light on the nuanced psychological and behavioral dynamics involved in pre-race nutrition among international runners. By examining their inner dialogues, anticipatory anxieties, and coping strategies, it becomes evident that the act of eating prior to competition involves complex processes of emotional regulation and the management of personal identity. The findings underscore that nutritional decisions in the context of sports tourism are deeply embedded in personal histories, perceived risks, and shaped notions of control and preparation.

The first theme, anticipatory anxiety, highlights that food-related decisions prior to competition are strongly influenced by preemptive stress and cognitive over-engagement. While sports nutrition literature emphasizes the optimization of macronutrient intake and timing for performance enhancement [46,47,48,49], this study shows that such decisions are frequently overshadowed by worry and perceived vulnerability. Participants described a psychological balance, where a single nutritional misstep was believed to threaten not only physical performance but also emotional stability. This aligns with findings from sport psychology that emphasize the anticipatory nature of competitive stress [50,51,52], and suggests that nutritional uncertainty may operate as a discrete domain of performance anxiety in the context of endurance sport tourism.

The second theme, internal negotiation between nutritional knowledge and situational trust, illustrates a dissonance between athletes’ established dietary schemas and the dynamic environments they encounter while traveling. Participants often possessed sound knowledge of sports nutrition principles but hesitated to apply them in unfamiliar cultural contexts. This suggests that nutrition-related behavior is not merely a function of informational literacy but also of environmental confidence. Known from economics, theories of bounded rationality [53,54] may offer insight here, as athletes revert to heuristics or emotionally guided judgments when navigating uncertain or foreign foodscapes. This finding resonates with previous tourism research, where food neophobia and perceptions of hygiene have been shown to influence dietary choices abroad [55,56,57,58].

Interestingly, participants’ food choices were not always governed by health optimization alone but reflected a psychological calculus that weighed familiarity, safety, and emotional comfort against theoretical correctness. This reveals a pragmatic and affective dimension to nutritional decision-making, where emotional regulation is embedded within the consumption process itself [59]. Rather than treating deviations from normative sports nutrition practices as lapses, these behaviors should be understood as adaptive responses to situational constraints and internal emotional demands.

The third theme, ritualization and individualized norms as fundamental mechanisms of psychological regulation, reveals the protective role of individual routines and symbolic practices. Pre-race rituals, including specific foods, timings, or sources, act as psychological stabilizers that help mitigate perceived chaos. These rituals can be seen through the lens of Giddens’ theory of ontological security [60], where repeated practices provide a sense of order in a complex and uncertain environment. In addition, drawing on anthropological theories of liminality [61,62] and contemporary work on performative rituals in sport [63], these findings suggest that such behaviors offer emotional continuity and perceived stability amidst the volatility of travel and competition. Importantly, the symbolic function of food—as a means of imposing personal structure onto the unpredictable space of international travel and race preparation—extends the conceptualization of eating practices beyond mere nutritional necessity and into the domain of emotional self-regulation.

The psychological and behavioral dynamics observed in this study can be further contextualized through several foundational theories in health psychology and stress regulation. The Health Belief Model [64] helps to explain how athletes framed food decisions in terms of risk and vulnerability. For instance, the theme of anticipatory anxiety revealed that runners often viewed nutrition as a domain of potential threat, where even a small mistake could jeopardize performance (cf. R1, R4). Concerns about safe choices and the avoidance of unfamiliar meals (R8, R10) reflect perceptions of susceptibility and severity, while careful planning of meals in advance highlights a preventive orientation. Similarly, the Theory of Planned Behavior [65] sheds light on the role of intention and perceived control. Athletes voiced clear plans to follow established routines, but these intentions were frequently challenged by situational constraints such as limited food options while traveling (R6, R9), illustrating the tension between rational planning and contextual demands. Lazarus’s transactional model of stress, appraisal, and coping [66] further clarifies how food-related stress was continuously appraised and re-appraised. Avoidance of unknown foods can be understood as a primary appraisal strategy aimed at reducing perceived threat (R2, R6), whereas flexible adjustments—such as negotiating available options or adapting pre-prepared plans (R4, R8)—demonstrate secondary coping strategies that restored a sense of control. This duality between avoidance and adaptation shows how food-related stress was regulated dynamically in response to the competitive context. At last, Self-Determination Theory [67] underscores how autonomy, competence, and relatedness shaped the runners’ nutritional behavior. The theme of autonomy emerged in participants’ reflections on trusting their own body’s signals despite situational constraints (R6, R10). Feelings of competence were evident when athletes linked routine eating habits and rituals with readiness and confidence (R2, R11). Meanwhile, relatedness appeared more subtly in shared practices of avoiding surprises while traveling and in the comfort derived from familiar meals (R8, R3). These findings suggest that pre-race nutrition was not only about physiological preparation but also about sustaining intrinsic motivation, emotional stability, and psychological well-being in an uncertain environment.

These findings contribute to a broader discussion on the interdependence between tourism, sport, and food behavior. While sports tourism literature has primarily focused on motivations, identity, and travel logistics [68,69,70,71], this study positions nutrition as a key psychosocial domain that intersects with all these elements. Unlike leisure tourists, athletes often have competing demands: the desire to explore and the need to control, the pressure to perform and the wish to enjoy. Food serves as both the focal point and the mechanism through which these competing demands are negotiated. This duality—between exploration and restraint—is particularly acute in endurance sport contexts, where bodily readiness is paramount.

Moreover, the study’s focus on inner dialogues draws attention to the largely underexplored intrapersonal dimension of nutritional behavior, which has previously been addressed in a markedly different scope by Kimiecik and Horn [72]. The runners participating in this study demonstrate that anxiety related to undernourishment is not always visible or explicitly articulated, yet it profoundly influences behavior. Recognizing this subjective experience opens the door to more holistic support strategies that integrate physiological, psychological, and cultural competencies.

## 5. Study Limitations and Practical Implications

When interpreting the findings of this study, several limitations should be considered. The qualitative methodology, relying mainly on participants’ self-reported experiences, introduces a degree of subjectivity that may influence how nutritional decisions and perceptions were expressed. Personal biases, memory recall limitations, and unconscious influences might not have been fully accounted for, potentially affecting the depth and reliability of the insights. Moreover, the relatively small and homogeneous sample—consisting only of runners from Ukraine, Germany, and the United Kingdom—limits the diversity of perspectives and reduces the generalizability of the findings, even within the qualitative research framework. The study did not examine potential gender-specific or age-related patterns in nutritional stress, as the sample size and qualitative focus did not allow for meaningful subgroup comparisons. Furthermore, key variables such as experience level and personality traits of participants were not assessed, which could have influenced nutritional decision-making and coping strategies. Additionally, the research focused solely on participants of the Poznan Half Marathon, meaning that local conditions and event-specific factors may have influenced the nutritional choices and experiences shared by the athletes, further restricting broader applicability. The lack of quantitative data prevents an evaluation of the prevalence and statistical relationships of particular nutritional behaviors or attitudes, which could have enhanced the study’s rigor. Finally, various psychophysical and environmental factors, such as individual health status, training load, and other situational variables, were not addressed but may have a significant impact on runners’ nutritional needs and perceptions. It is important to emphasize that this study had an exploratory aim, focusing on uncovering complex psychosocial dynamics and internal dialogues around pre-race nutrition rather than establishing causal relationships. Future studies incorporating these elements would contribute to a more holistic understanding of nutrition within the framework of international sport tourism.

To effectively reduce nutritional anxiety, organizers could implement targeted interventions such as pre-race workshops or counseling sessions designed to prepare athletes for food-related challenges in foreign environments. Providing a variety of familiar, reliable food options alongside well-communicated local alternatives can help alleviate anticipatory stress and boost athletes’ sense of control. Moreover, personalized nutritional rituals deserve particular attention; encouraging runners to maintain or adapt their routines respects individual differences and fosters emotional well-being. Practical tools such as multilingual guides detailing local cuisine, allergen information, and trusted food vendors can empower athletes to make informed dietary choices, reducing uncertainty and risk. Innovative solutions like tailored meal plans or nutrition kits specifically designed for athletes can ease the stress of pre-race nutrition and contribute to a more positive overall experience. Integrating these strategies into race-day planning not only supports physical readiness but also enhances psychological comfort, ultimately improving athlete satisfaction and potentially boosting performance.

## 6. Conclusions

This study sheds light on the complex, often invisible psychological processes that shape pre-race nutritional behaviors among runners participating in international sporting events. While the findings reveal important aspects of athletes’ intrapersonal experiences, the study does not claim that these inner dialogues are central determinants of sport nutrition or performance psychology outcomes. However, far from being a purely physiological or logistical matter, eating in the pre-competition phase emerges as a deeply intrapersonal experience—one in which food choices are entangled with anticipatory anxiety, internal negotiations, and personalized rituals of control. The findings reveal that these athletes do not merely eat to fuel performance; they eat to manage fear, assert autonomy, and stabilize their inner world amidst the uncertainties of travel and competition. In particular, the repetitive use of individualized rules and dietary routines functions not only as a strategy for minimizing risk, but also as a subtle form of emotional self-regulation. By highlighting the inner dialogues that precede nutritional decisions, this research invites a broader, more integrated understanding of sports nutrition—one that encompasses not just bodily needs, but also psychological safety, identity, and agency. While further research is needed to establish the direct impact of these psychological processes on athletic performance, such an approach may inform more nuanced support systems that bridge the physiological, psychological, and cultural dimensions of athletic preparation in transnational contexts.

## Figures and Tables

**Table 1:** Table of participants.

Alias	Gender	Country of Residence
R1	Male	United Kingdom
R2	Male	United Kingdom
R3	Male	United Kingdom
R4	Female	United Kingdom
R5	Male	Germany
R6	Male	Germany
R7	Male	Germany
R8	Female	Germany
R9	Male	Ukraine
R10	Male	Ukraine
R11	Female	Ukraine
R12	Female	Ukraine

## Data Availability

The original contributions presented in the study are included in the article; further inquiries can be directed to the corresponding author.
